# *De novo* transcriptome assembly databases for the butterfly orchid *Phalaenopsis equestris*

**DOI:** 10.1038/sdata.2016.83

**Published:** 2016-09-27

**Authors:** Shan-Ce Niu, Qing Xu, Guo-Qiang Zhang, Yong-Qiang Zhang, Wen-Chieh Tsai, Jui-Ling Hsu, Chieh-Kai Liang, Yi-Bo Luo, Zhong-Jian Liu

**Affiliations:** 1 State Key Laboratory of Systematic and Evolutionary Botany, Institute of Botany, Chinese Academy of Sciences, Beijing 100093, China; 2 University of Chinese Academy of Sciences, Beijing 100049, China; 3 Shenzhen Key Laboratory for Orchid Conservation and Utilization, The National Orchid Conservation Centre of China and The Orchid Conservation and Research Centre of Shenzhen, Shenzhen 518114, China; 4 Institute of Tropical Plant Sciences, National Cheng Kung University, Tainan 701, Taiwan; 5 Orchid Research and Development Center, National Cheng Kung University, Tainan 701, Taiwan; 6 Department of Life Sciences, National Cheng Kung University, Tainan 701, Taiwan; 7 The Centre for Biotechnology and BioMedicine, Graduate School at Shenzhen, Tsinghua University, Shenzhen 518055, China; 8 College of Forestry and Landscape Architecture, South China Agricultural University, Guangzhou 510640, China; 9 College of Arts, College of Landscape Architecture, Fujian Agriculture and Forestry University, Fuzhou 350002, China

**Keywords:** High-throughput screening, Transcriptomics, Molecular evolution

## Abstract

Orchids are renowned for their spectacular flowers and ecological adaptations. After the sequencing of the genome of the tropical epiphytic orchid *Phalaenopsis equestris*, we combined Illumina HiSeq2000 for RNA-Seq and Trinity for *de novo* assembly to characterize the transcriptomes for 11 diverse *P. equestris* tissues representing the root, stem, leaf, flower buds, column, lip, petal, sepal and three developmental stages of seeds. Our aims were to contribute to a better understanding of the molecular mechanisms driving the analysed tissue characteristics and to enrich the available data for *P. equestris*. Here, we present three databases. The first dataset is the RNA-Seq raw reads, which can be used to execute new experiments with different analysis approaches. The other two datasets allow different types of searches for candidate homologues. The second dataset includes the sets of assembled unigenes and predicted coding sequences and proteins, enabling a sequence-based search. The third dataset consists of the annotation results of the aligned unigenes versus the Nonredundant (Nr) protein database, Kyoto Encyclopaedia of Genes and Genomes (KEGG) and Clusters of Orthologous Groups (COG) databases with low e-values, enabling a name-based search.

## Background & Summary

Orchidaceae is the most diverse family of angiosperms, including approximately 25,000 species (i.e., approximately 8% of all vascular plant species), more than mammals, birds and reptiles combined^1^. Orchids are known for their very diverse and specialized reproductive and ecological strategies. The specific development of the labellum (the ‘lip’) and gynostemium (a fused structure of the stamens and pistils) to trick pollinators and to facilitate pollination is well documented^2,3^. In addition to the highly sophisticated floral structure, crassulacean acid metabolism (CAM), symbiosis with fungi, and epiphytism might also be linked to the adaptive radiation of orchids^4–6^, which might be related to their high diversification. Since the publication of Charles Darwin’s book *On the Origin of Species*, evolutionary biologists have been fascinated by orchids. Biologists have proposed various explanations for the extraordinary diversity of orchids but have been unable to identify its root causes.

The genome sequence of the tropical epiphytic orchid *Phalaenopsis equestris*, which represents the first sequenced genome for a plant with CAM, contains 29,431 predicted protein-coding genes^3^. The genomic sequence shows evidence of an orchid-specific polyploidy event that preceded the radiation of most orchid clades and suggests that gene duplication might have contributed to the evolution of CAM photosynthesis in *P. equestris*^3^. In addition, this species possesses expanded and diversified families of MADS-box C/D-class, B-class AP3, and AGL6-class genes, which might contribute to the highly specialized morphology of orchid flowers^3^. Furthermore, the *P. equestris* genome does not contain the β group of type I MADS-box genes (type I Mβ), although these genes do exist in *Arabidopsis thaliana*, *Populus trichocarpa,* and *Oryza sativa*. Interactions among type I MADS-box genes are important for the initiation of endosperm development^7^.

Some cDNA libraries have been constructed to examine the gene expression in *Phalaenopsis* mature flower buds^8^ and floral scent products by comparing their expression patterns in *P. bellina* and in the scentless species, *P. equestris*^9^, for which expressed sequence tags (ESTs) were sequenced and assembled into unigenes. *Phalaenopsis* ESTs are derived from cDNA-amplified fragment length polymorphism (cDNA-AFLP) and randomly amplified polymorphic cDNAs (cDNA-RAPD)^10,11^. These methods were used to systematically screen many differentially expressed cDNA fragments in the wild-type strain and somaclonal variants^10,11^. Several differentially expressed transcripts related to flower development and flower colour were identified^10,11^.

Two orchid transcriptomic databases have been established. One is OrchidBase, which contains the transcriptome sequences derived from 11 *Phalaenopsis* orchid cDNA libraries. OrchidBase was constructed from different species, including *P. Aphrodite* subsp. formosana, *P. equestris* and *P. bellina*, and from different tissues, including the developing seed, protocorm, vegetative tissue, leaf, cold-treated plantlet, pathogen-treated plantlet, inflorescence and flower buds^12,13^. The other database, Orchidstra, was constructed from the 233,924 unique contigs of the transcriptome sequences of *P. aphrodite* subsp. *formosana*. In Orchidstra, genes with tissue-specific expression were categorized by profiling analysis with RNA-Seq^14^.

Recently, the first comprehensive analysis of the transcriptome and expression profiles during *Phalaenopsis* explant browning was reported using Illumina high-throughput technology. In this genome-wide level analysis, differentially expressed genes (DEGs) before and after *Phalaenopsis* explant browning were identified^15^. In addition, to study the regulation of *Phalaenopsis* flower organ development, RNA-Seq reads were generated with the Illumina platform for floral organs of the *Phalaenopsis* wild-type strain and a peloric mutant with a lip-like petal. In total, 43,552 contigs were obtained after *de novo* assembly. The comprehensive transcript profile and functional analysis suggest that *PhAGL6a*, *PhAGL6b* and *PhMADS4* might play crucial roles in *Phalaenopsis* labellum development^16^.

**Figure 1:**
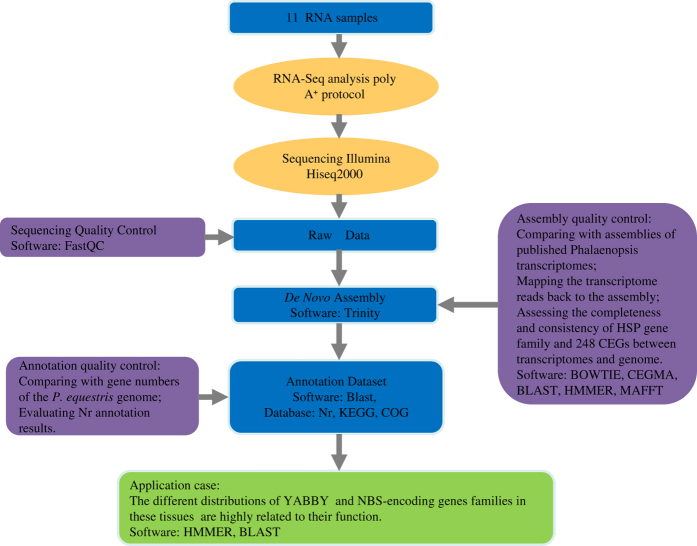
Schematic overview of the study. We collected one sample for each tissue type, including root, stem, leaf, flower bud, column, lip, petal, sepal and seeds from three developmental stages of *P. equestris*. Next, we sequenced cDNAs generated from the tissues on an Illumina HiSeq2000 in 90-bp paired-end (PE) reads, with 75-bp paired-end (PE) reads from the leaf tissue. The analysis started with assembling the short reads using the *de novo* assembly program Trinity and continued with functional analysis using BLASTX. Moreover, we performed quality control assessments at each step from the raw reads to the annotation datasets. Finally, we used YABBY and NBS-encoding gene families as examples of the usage of these datasets.

All this genomic and transcriptomic information will supply datasets for orchid molecular biology research. Here, we chose to focus on the transcriptomes of the root, stem, leaf, flower buds, column, lip, petal, sepal and three developmental stages of seeds from an individual plant of *P. equestris* used for genome sequencing. We provided high-quality transcriptome assemblies and annotated results, enabling comparisons with previously generated *Phalaenopsis* transcriptome data from the same or different tissues to further understand the highly specialized morphology of orchid flowers and the adaptive radiation of this highly diverse plant group. We also first presented the usage of these datasets using YABBY and NBS-encoding gene families as examples. All the experimental processes involved in the paper are shown in Fig. 1.

## Methods

These methods are expanded from descriptions previously published in *Nature Genetics*^3^.

### Plant sample collection and conditions

The experiments were performed on nine butterfly orchid *P. equestris* tissues: root, stem, leaf, flower buds, column, lip, petal, sepal and three developmental stages of seeds. All these tissues were obtained from the adult plant that was also used for genome sequencing and were grown at the National Orchid Conservation Centre of China and stored at −80 °C for further experiments.

### Experimental design

One sample of each tissue of *P. equestris*—root, stem, leaf, flower buds, column, lip, petal, sepal and three developmental stages of seeds—was taken for RNA sequencing. The stem without the bud was from a three-year-old plant. The seeds we used for RNA sequencing were sown on 1/2 Murashige-Skoog (MS) medium for 4, 7 and 12 days, respectively.

### RNA collection

**Figure 2:**
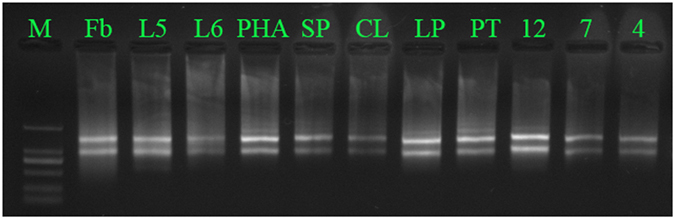
RNA from eleven tissues analysed by agarose gel electrophoresis. CL, column; Fb, flower bud; L5, root; L6, stem; LP, lip; M, Marker DL2000; PHA, leaf; PT, petal; SP, sepal; 12, 12-day seed; 7, 7-day seed; 4, 4-day seed.

Total RNA was extracted from each tissue using an RNAprep Pure Plant Kit (Qiagen). The quality and quantity of each RNA sample was assessed by agarose gel electrophoresis (Fig. 2).

### Library construction and illumina sequencing

A total of 3 μg RNA per sample was used to construct the cDNA library. Poly(A) mRNA was purified from total RNA using oligo(dT)-attached magnetic beads. Fragmentation buffer was used to cleave the mRNA into short fragments, which were then used as templates for the random hexamer-primed synthesis of first-strand cDNA. Second-strand cDNA was synthesized using buffer, dNTPs, RNase H, and DNA polymerase I. From this cDNA, a paired-end library was synthesized using a Genomic DNA Sample Preparation Kit (Illumina), according to the manufacturer’s instructions. Short fragments were purified with a QIAquick Gel Extraction Kit (Qiagen) and were then resolved with EB buffer for end repair and the addition of poly(A) tails. The short fragments were then connected with sequencing adapters, and suitable fragments were separated by agarose gel electrophoresis. Finally, the sequencing library was constructed by PCR amplification, and eleven cDNA libraries were generated. Sequencing using the Illumina HiSeq2000 system was performed to generate 90-bp paired-end (PE) reads, except in the leaf, for which 75-bp paired-end reads were generated.

### *De novo* assembly and dataset annotation

*De novo* transcriptome reconstruction was performed using Trinity (version trinityrnaseq-r2013-02-25)^17^. Trinity was applied using the inchworm method with a minimum contig length of 200 nucleotides. The default settings for Trinity paired-end assembly were used for the remaining parameters. The assembly was further spliced and assembled to acquire non-redundant unigenes that were as long as possible. BLASTX (e-value≤1e^−5^) was performed to annotate the unigenes based on protein databases, including Nr, KEGG, and COG. The CDSs (coding DNA sequences) and protein sequences of all unigenes were predicted using BLASTX, ESTScan^18^, and the fifth-order Markov model. First, we utilized protein databases such as Nonredundant (Nr), Kyoto Encyclopaedia of Genes and Genomes (KEGG), and Clusters of Orthologous Groups (COG) to align against the unigenes using BLASTX with an E-value cutoff of 1e^−5^. The best alignment results were used to determine the sequence directions of the unigenes. Unigenes with sequences that produced matches in only one database were not searched further. When a unigene would not align to any database, ESTScan was used to predict coding regions and to determine the sequence direction. If the above two methods still could not predict the CDSs of the unigenes, we used a fifth-order Markov model to predict the CDSs.

### HSP90, HSP70 and YABBY gene family identifications

We used hmmsearch of the Hidden Markov Model (HMM)-based HMMER program (3.3.2)^19^ to identify all HSP90, HSP70 and YABBY genes. HMM profiles of the HSP90, HSP70 and YABBY gene families (PF00183, PF00012 and PF04690.8 in pfam database^20^) were used in local searches of the *P. equestris* (PEQU) database, and deposited to Dryad Digital Repository (Data Citation 1). Subsequently, we used the Blastp program to search for the HSP90, HSP70 and YABBY genes in these transcriptomic protein datasets using the protein sequences of individual putative *P. equestris* HSP90, HSP70 and YABBY as queries, respectively. To maximize the confidence, only the HSP90 and HSP70 genes with E-values of 0.0 and YABBY genes with E-values ≤1e^−20^ were chosen, filtered for perfect matches, and aligned using MAFFT^21^ with an E-INS-I alignment strategy for sequence integrity analysis.

### Identification of NBS-encoding genes

The complete set of NBS-encoding sequences was identified from the *P. equestris* genome^3^ in a reiterative process. First, all predicted proteins from the annotation of the *P. equestris* genome were screened using HMMER V.3 (ref. 19) analysis against the raw HMM corresponding to the Pfam NBS (NB-ARC) family (PF00931). The raw NB-ARC HMM was downloaded from the Pfam home page (http://pfam.xfam.org/)^20^. A set of 58 genes with the NBS motif was selected from the HMM search results with E-values ≤1e^−10^. In the second analytical step, selected protein sequences were aligned based only on the NBS domain using Muscle^22^. Next, the alignment was used to construct a *P. equestris*-specific HMM model. The refined HMM was compared against all predicted proteins from the *P. equestris* genome, and 65 genes were identified. In the third step, the NBS domains of the 65 identified genes were incorporated into the refined HMM model, which was used to search for related sequences. We also identified the same 65 genes in this step, which indicated that those 65 genes were reliable NBS-encoding gene candidates. Then, we used the Blastp program to search for NBS-encoding genes in these transcriptomic datasets against those 65 genes. To maximize the confidence, the NBS-encoding genes were further confirmed using SMART (http://smart.embl-heidelberg.de/).

### Genome annotation methods

The methods for *P. equestris* genome assembly and annotation (Table 1) were presented in the previous publication^3^.

## Data Records

For this study, we deposited six datasets. The first dataset consists of the genome annotation, constructed library reads and assembly sequences of *P. equestris* (Data Citations 1 and 2 and Tables 1–3). The genome annotation and scaffolds were deposited to the Dryad Digital Repository (Data Citation 1 and Table 1), while the 37 DNA paired-end library data, contigs and scaffolds were submitted to the NCBI database (Data Citation 2 and Tables 2 and 3). The second dataset consists of the RNA-Seq raw reads. This dataset contains a total of eleven samples (Data Citation 3 and Table 4). The third dataset contains the unigenes of the eleven samples (Data Citation 1 and Table 5). The fourth dataset is the annotation file, which contains the results annotated using all three databases and the predicted CDSs and protein files from the results from the eleven tissues (Data Citation 1 and Table 6 (available online only)). The fifth dataset includes the aligned full-length sequences of the HSP90 and HSP70 gene families, showing the integrity of the assembly (Data Citation 1 and Table 7). The sixth dataset contains the alignment results from 100 randomly selected conserved core eukaryotic genes (CEGs) among *Arabidopsis thaliana, P. equestris* and eleven transcriptomes for examining the transcript assembly completeness (Data Citation 1). The first dataset described above (Data Citations 1 and 2 and Tables 1–3) was previously published in our related work in the journal *Nature Genetics*^3^. The second dataset (Data Citation 3 and Table 4), the third dataset (Data Citation 1 and Table 5) and the fourth dataset (Data Citation 1 and Table 6 (available online only)) are the core of this work and have not been published previously.

## Technical Validation

### Sequencing quality control

We used two steps for testing sequencing quality. The first step included counts of the total reads and total bases for each sample to ensure that the amounts were approximately of the same order of magnitude. These amounts were 16–70 million reads (Table 8). As a second step, we tested samples using FastQC^23^ for Q20 and GC content (Table 8).

### Assembly quality control

To ensure that the produced contigs were correct following the use of Trinity, we compared our transcriptome model to the published *Phalaenopsis* transcriptomes. We compared basic statistics, such as the average contig length (Table 9), which was longer than the average transcript size from OrchidBase^13^ (approximately 350 bp, http://orchidbase.itps.ncku.edu.tw/), and shorter than those from leaves of *Phalaenopsis* sp. (ref. 15) and *Phalaenopsis* Brother Spring Dancer ‘KHM190’ (ref. 16), 1,108.07 and 2,094, respectively. We also compared the total transcripts with the number of those mapped to the *P. equestris* genome^3^, which has a similar number. We subsequently tested full-length transcripts against the HSP90 gene family^24^ to examine the completeness of the data. We found only one gene (*Unigene017669_ORF*) in the leaf (PHA), one gene (*Unigene037471_ORF*) in the root (L5), and one gene (*Unigene029033_ORF*) in the flower bud (fb) that were almost full-length; the others were reconstructed perfectly (the fifth dataset in Data Citation 4). We also found that there was partial sequencing missed in the PEQU_19561 gene of *P. equestris* genome. We also tested the HSP70 gene family, which is constitutively expressed and up-regulated in response to various stressors, such as heat, cold, anoxia, and heavy metal exposure^25,26^. Only six pairs of unigenes should be merged based on the sequence analysis: *Unigene019149_ORF* and *Unigene019150_ORF* in the fb, *Unigene052632_ORF* and *Unigene052633_ORF* in the stem (L6), and *Unigene020433_ORF* and *Unigene020432_ORF* in the PHA, *sepal_c24932_g1_i1_7684* and *sepal_c24932_g2_i1_6884* in the sepal, *petal_c31129_g2_i1_17690* and *petal_c31129_g1_i1_15744* in the petal, *column_c50529_g2_i1_17726* and *column_c50529_g1_i1_29153* in the column. In addition, two genes of the *P. equestris* genome had missed sequences: *PEQU_21700* and *PEQU_20114* (the fifth dataset in Data Citation 1). The HSP70 sequences from the root, lip and three developmental stages of seeds were perfectly reconstructed (the fifth dataset in Data Citation 1). Next, we used Bowtie to map the reads back to the unigenes to test the mapping rate (Table 10)^17^; more than 85% of the reads were proper pairs, showing a high read utilization rate. Finally, the 248 conserved CEGs were used to assess transcript assembly completeness using CEGMA software^27^ (Table 10). The completeness of PHA was likely low because fewer reads were returned or because some conserved CEGs are not expressed. The transcript assembly completeness of all other tissues had high values (i.e., greater than 80%). We manually examined 100 randomly selected CEG sequences from *A. thaliana* to align with PEQU genome sequences and eleven tissue transcriptome homologous genes (the sixth dataset in Data Citation 1). Of these, 82 CEG sequences (82%) were perfectly reconstructed, showing high consistency, although some sequences suggested that partial sequencing was missed in the PEQU genome, such as sequences from At2g36880.1 homologous genes, and some sequences in transcriptomes should be merged, such as sequences from At4g39280.1 homologous genes.

### Annotation quality control

**Figure 3:**
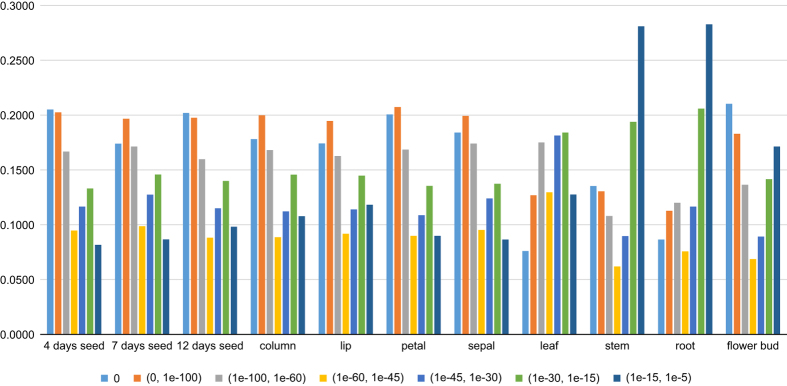
E-value distribution of the blast results for the eleven transcriptome unigenes in the Nr database. The x-axis shows the eleven tissues, different colours outline the range of E-values, and the y-axis provides the percentages.

We estimated the functional annotation results based on the aforementioned database and detailed information from the Nr database (Table 11 and Fig. 3), which revealed 18,787–32,996 unigenes with low e-values that were aligned versus the Nr database showing similar annotation gene numbers with the *P. equestris* genome^3^. Additionally, the statistical results of the predicted CDSs are shown in Table 12.

## Usage Notes

The data provided in these experimental datasets can be used for the following two purposes. First, it is possible to use the raw reads to conduct new experiments using different analytical methods. Second, each analysis step can be performed differently because all the technical experimental information is publicly available.

### *De novo* assembly

Using the unigenes generated with Trinity, a dataset search for genes of interest can be easily performed by searching for homologues using Blast or by performing a text-based search when using an annotation table. We can also identify the gene families that are expressed in specific tissues. For example, we demonstrated that the YABBY gene family plays a key role in determining leaf polarity^28–30^. The results indicated that the gene family does not exist in the root and seed (Table 13), a finding that is consistent with their function. Furthermore, we identified disease resistance (*R*) genes (Table 14), which play important roles in resistance to major plant pathogens^31^, and NBS domain sequences that are commonly used to identify *R* genes and to classify the genes into subgroups bearing different functions^32^. Among these tissues, at least 7 *R* genes were identified in the 7-day seeds, whereas 24, 21, and 22 genes were found in the flower bud, root and stem, respectively. These findings suggest that the flower bud, stem and root may be more susceptible than 7-day seeds to major diseases or that resistance to various orchid pathogens is related to not only the *R* gene numbers but also *R* gene expression. We also found that MADS-box genes mostly existed in flower tissue, suggesting a distinct role for these genes in orchid floral morphogenesis^3^.

### Downstream analysis

Future downstream analyses could entail a comparison of the tissues sequenced in this work to other tissues to determine genes that are differentially expressed in other plant organs. Additionally, because orchids are divided into different ecotypes (epiphytic, lithophytic, and terrestrial plants)^5,33^, comparing transcriptomes from the same tissues, particularly the root, among different orchid ecotypes could provide new insights into the molecular mechanisms of orchid ecological differentiation.

## Additional Information

**How to cite this article:** Niu, S.-C. *et al.* *De novo* transcriptome assembly databases for the butterfly orchid *Phalaenopsis equestris*. *Sci. Data* 3:160083 doi: 10.1038/sdata.2016.83 (2016).

## Supplementary Material



## Figures and Tables

**Table 1:** Genome sequences of the *P. equestris* deposit.

**File name**	**File type**	**Data description**
*Scaffolds*		
Pha_1213.scafSeq.FG2_superscaffold	fasta	Genome assembly results file
Pha_1213.scafSeq.FG2_superscaffold.link	txt	File containing the locational relationship between superscaffold and scaffolds or contigs
*Repeat*		
Pha_1213.scafSeq.FG2.Proteinmask.annot.known.trans.fa	fasta	Repeat annotation file by proteinmasker
Pha_1213.scafSeq.FG2.Proteinmask.annot.known.trans.gff	gff	gff file of repeat annotation by proteinmasker
Pha_1213.scafSeq.FG2.RepeatMasker.out.known.trans.fa	fasta	Repeat annotation file by repeatmasker
Pha_1213.scafSeq.FG2.RepeatMasker.out.known.trans.gff	gff	gff file of repeat annotation by repeatmasker
Pha_1213.scafSeq.FG2.denovo.trans.gff	gff	*De novo* repeat annotation gff format file
Pha_1213.scafSeq.FG2.trf.out.known.tran.fa	fasta	Repeat annotation file by TRF
Pha_1213.scafSeq.FG2.trf.out.known.tran.gff	gff	gff file of repeat annotation by TRF
repeat_statistics.xlsx	xlsx	statistics of repeat annotation
*Gene models*		
P.equestis.gene.cds	fasta	Predicted coding sequence
P.equestis.gene.gff	gff	Annotated coding sequence, gff format file
P.equestis.gene.pep	fasta	Predicted protein sequence
*Function annotation*		
Interpro.tar	tar	InterPro database annotation
KEGG.tar	tar	KEGG database annotation
Swissprot.tar	tar	Swissprot database annotation
Trembl.tar	tar	TrEMBL database annotation

**Table 2:** Summary of the construction of the 37 libraries deposited in the NCBI database.

**Run**	**MBases**	**MBytes**	**Experiment**	**Insert Size**
SRR827602	3,332	2,288	SRX265492	344
SRR827603	3,255	2,233	SRX265493	335
SRR827604	2,635	1,814	SRX265494	800
SRR827605	2,612	1,831	SRX265495	800
SRR827606	2,875	1,998	SRX265496	800
SRR827607	2,903	2,007	SRX265496	800
SRR827608	2,895	1,990	SRX265496	800
SRR827609	2,867	1,966	SRX265496	800
SRR827610	2,586	1,812	SRX265497	335
SRR827611	2,531	1,765	SRX265497	335
SRR827612	2,464	1,711	SRX265497	335
SRR827613	2,941	2,025	SRX265498	344
SRR827614	2,902	2,018	SRX265498	344
SRR827615	2,935	2,040	SRX265498	344
SRR827616	2,648	1,861	SRX265499	800
SRR827617	2,606	1,828	SRX265499	800
SRR827618	2,631	1,845	SRX265499	800
SRR827619	2,600	1,825	SRX265499	800
SRR827620	5,872	2,648	SRX265500	163
SRR827621	2,681	1,013	SRX265501	5000
SRR827622	2,372	854	SRX265502	5000
SRR827623	2,430	881	SRX265503	2000
SRR827624	2,535	947	SRX265504	2000
SRR827625	2,432	956	SRX265505	2000
SRR827626	2,632	1,002	SRX265506	2000
SRR827627	2,375	847	SRX265507	5000
SRR827628	12,673	7,935	SRX265508	163
SRR827629	15,710	8,791	SRX265509	163
SRR827630	14,766	9,139	SRX265510	163
SRR827631	3,089	1,669	SRX265511	20000
SRR827632	5,125	2,829	SRX265512	10000
SRR827633	6,567	3,440	SRX265513	20000
SRR827634	6,260	3,239	SRX265514	10000
SRR827635	7,762	3,960	SRX265515	2000
SRR827636	8,168	4,209	SRX265516	5000
SRR827637	5,656	3,580	SRX265517	40000
SRR827638	5,708	3,615	SRX265518	40000

**Table 3:** Global genome assembly statistics deposited in the NCBI database.

Total sequence length	1,064,051,384
Total assembly gap length	80,500,320
Number of scaffolds	89,583
Scaffold N50	378,442
Scaffold L50	493
Number of contigs	188,397
Contig N50	21,144
Contig L50	12,818

**Table 4:** Raw data deposit.

**Sample no.**	**SRA Runs**	**BioSample**	**Title**
1	SRR2080194	SAMN03799292	Phalaenopsis_equestris_root_RNA_Seq_fastq_files
2	SRR2080204	SAMN03799301	Phalaenopsis_equestris_flower_RNA_Seq_fastq_files
3	SRR2080202	SAMN03799299	Phalaenopsis_equestris_leaf_RNA_Seq_fastq_files
4	SRR2080200	SAMN03799297	Phalaenopsis_equestris_stem_RNA_Seq_fastq_files
5	SRR3606718	SAMN05185248	Phalaenopsis equestris seed 12 days RNA_seq fastq files
6	SRR3606742	SAMN05185247	Phalaenopsis equestris seed 7 days RNA_seq fastq files
7	SRR3606734	SAMN05185246	Phalaenopsis equestris seed 4 days RNA_seq fastq files
8	SRR3602300	SAMN05185245	Phalaenopsis equestris sepal RNA_seq fastq files
9	SRR3602299	SAMN05185244	Phalaenopsis equestris petal RNA_seq fastq files
10	SRR3602277	SAMN05185243	Phalaenopsis equestris lip
11	SRR3600816	SAMN05185242	Phalaenopsis equestris column
This dataset contains 11 total samples. Sample 1 is from the root of *P. equestris*, sample 2 is from the flower buds of *P. equestris*, sample 3 is from the leaf of *P. equestris*, sample 4 is from the stem of *P. equestris,* samples 5, 6 and 7 are seeds sown on 1/2 MS medium for 12, 7 and 4 days, and samples 8, 9, 10 and 11 are the sepal, petal, lip and column, respectively. The sequenced data were deposited in the Sequence Read Archive (SRA, accession numbers SRR2080194, SRR2080204, SRR2080202, SRR2080200, SRR3606718, SRR3606742, SRR3606734, SRR3602300, SRR3602299, SRR3602277, and SRR3600816) (Data Citation 3).			

**Table 5:** Unigene deposit.

**File name**	**File type**	**Data**
fb.Unigene.fa	fasta	unigene
L5.Unigene.fa	fasta	unigene
L6.Unigene.fa	fasta	unigene
PHA.Unigene.fa	fasta	unigene
12_day.unigene.fasta	fasta	unigene
7_day.unigene.fasta	fasta	unigene
4_day.unigene.fasta	fasta	unigene
sepal.unigene.fasta	fasta	unigene
petal.unigene.fasta	fasta	unigene
lip.unigene.fasta	fasta	unigene
colum.unigene.fasta	fasta	unigene
The dataset contains the unigenes from the longest contigs per transcripts generated using Trinity. The fb.Unigene.fa file contains unigenes from the flower bud of *P. equestris*, the L5.Unigene.fa file contains unigenes from the root of *P. equestris*, the L6.Unigene.fa file contains unigenes from the stem of P. equestris, and the PHA. Unigene.fa file contains unigenes from the leaf of *P. equestris*. The 12_day.unigene.fasta, 7_day.unigene.fasta and 4_day.unigene.fasta files are unigenes from seeds sown on 1/2 MS medium for 12, and 4 days. The sepal.unigene.fasta, petal.unigene.fasta, lip.unigene.fasta and colum.unigene.fasta files are unigenes from the sepal, petal, lip and column. The unigene files were deposited in the Dryad Digital Repository (Data Citation 1).		

**Table 6:** Annotation deposit.

**Transcriptome annotation**	**File name**	**File type**	**Data description**
fb. annotation	fb.blastx.cog.xls	xls	COG database annotation
	fb.blastx.kegg.xls	xls	KEGG database annotation
	fb.blastx.nr.xlsx	xlsx	Nr database annotation
	fb.cds	fasta	predicted coding sequence
	fb.pep	fasta	predicted protein sequence
L5. annotation	L5.blastx.cog.xls	xls	COG database annotation
	L5.blastx.kegg.xls	xls	KEGG database annotation
	L5.blastx.nr.xlsx	xlsx	Nr database annotation
	L5.cds	fasta	predicted coding sequence
	L5.pep	fasta	predicted protein sequence
L6. annotation	L6.blastx.cog.xls	xls	COG database annotation
	L6.blastx.kegg.xls	xls	KEGG database annotation
	L6.blastx.nr.xlsx	xlsx	Nr database annotation
	L6.cds	fasta	predicted coding sequence
	L6.pep	fasta	predicted protein sequence
PHA. annotation	PHA.blastx.cog.xls	xls	COG database annotation
	PHA.blastx.kegg.xls	xls	KEGG database annotation
	PHA.blastx.nr.xlsx	xlsx	Nr database annotation
	PHA.cds	fasta	predicted coding sequence
	PHA.pep	fasta	predicted protein sequence
4_day_seed_annotation	4_day_seed.blastx.cog.xls	xls	COG database annotation
	4_day_seed.blastx.kegg.xls	xls	KEGG database annotation
	4_day_seed.blastx.nr.xls	xls	Nr database annotation
	4_day_seed.cds	fasta	predicted coding sequence
	4_day_seed.pep	fasta	predicted protein sequence
7_day_seed_annotation	7_day_seed.blastx.cog.xls	xls	COG database annotation
	7_day_seed.blastx.kegg.xls	xls	KEGG database annotation
	7_day_seed.blastx.nr.xls	xls	Nr database annotation
	7_day_seed.cds	fasta	predicted coding sequence
	7_day_seed.pep	fasta	predicted protein sequence
12_day_seed_annotation	12_day_seed.blastx.cog.xls	xls	COG database annotation
	12_day_seed.blastx.kegg.xls	xls	KEGG database annotation
	12_day_seed.blastx.nr.xls	xls	Nr database annotation
	12_day_seed.cds	fasta	predicted coding sequence
	12_day_seed.pep	fasta	predicted protein sequence
column_annotation	column_day_seed.blastx.cog.xls	xls	COG database annotation
	column_day_seed.blastx.kegg.xls	xls	KEGG database annotation
	column_day_seed.blastx.nr.xls	xls	Nr database annotation
	column_day_seed.cds	fasta	predicted coding sequence
	column_day_seed.pep	fasta	predicted protein sequence
lip_annotation	lip_day_seed.blastx.cog.xls	xls	COG database annotation
	lip_day_seed.blastx.kegg.xls	xls	KEGG database annotation
	lip_day_seed.blastx.nr.xls	xls	Nr database annotation
	lip_day_seed.cds	fasta	predicted coding sequence
	lip_day_seed.pep	fasta	predicted protein sequence
sepal_annotation	sepal_day_seed.blastx.cog.xls	xls	COG database annotation
	sepal_day_seed.blastx.kegg.xls	xls	KEGG database annotation
	sepal_day_seed.blastx.nr.xls	xls	Nr database annotation
	sepal_day_seed.cds	fasta	predicted coding sequence
	sepal_day_seed.pep	fasta	predicted protein sequence
petal_annotation	petal_day_seed.blastx.cog.xls	xls	COG database annotation
	petal_day_seed.blastx.kegg.xls	xls	KEGG database annotation
	petal_day_seed.blastx.nr.xls	xls	Nr database annotation
	petal_day_seed.cds	fasta	predicted coding sequence
	petal_day_seed.pep	fasta	predicted protein sequence
The dataset contains functional annotations and gene coding sequence annotations for 11 tissues. There are five annotation files per tissue: three functional annotation files and two structural annotation files. The three functional annotation files are the COG, KEGG and Nr database annotation files. The.cds and.pep files are in fasta format; the titles in the files contain the unigene name predicted coding sequence, the locus and the coding direction. The annotation file was deposited in the Dryad Digital Repository (Data Citation 1).			

**Table 7:** HSP gene family deposit.

**File name**	**Data description**
hsp70_fb_PEQU.fas	alignment of the hsp70 genes from fb transcriptome and PEQU genome
hsp70_L5_PEQU.fas	alignment of the hsp70 genes from L5 transcriptome and PEQU genome
hsp70_L6_PEQU.fas	alignment of the hsp70 genes from L6 transcriptome and PEQU genome
hsp70_PHA_PEQU.fas	alignment of the hsp70 genes from PHA transcriptome and PEQU genome
hsp70_12_day_seed_pequ.fas	alignment of the hsp70 genes from 12 day seeds transcriptome and PEQU genome
hsp70_4_day_seed_pequ.fas	alignment of the hsp70 genes from 4 day seeds transcriptome and PEQU genome
hsp70_7_day_seed_pequ.fas	alignment of the hsp70 genes from 7 day seeds transcriptome and PEQU genome
hsp70_column_pequ.fas	alignment of the hsp70 genes from column transcriptome and PEQU genome
hsp70_lip_pequ.fas	alignment of the hsp70 genes from lip transcriptome and PEQU genome
hsp70_petal_pequ.fas	alignment of the hsp70 genes from petal transcriptome and PEQU genome
hsp70_sepal_pequ.fas	alignment of the hsp70 genes from sepal transcriptome and PEQU genome
hsp90_fb_PEQU.fas	alignment of the hsp90 genes from fb transcriptome and PEQU genome
hsp90_L5_PEQU.fas	alignment of the hsp90 genes from L5 transcriptome and PEQU genome
hsp90_L6_PEQU.fas	alignment of the hsp90 genes from L6 transcriptome and PEQU genome
hsp90_PHA_PEQU.fas	alignment of the hsp90 genes from PHA transcriptome and PEQU genome
hsp90_12_day_pequ.fas	alignment of the hsp70 genes from 12 day seeds transcriptome and PEQU genome
hsp90_4_day_pequ.fas	alignment of the hsp70 genes from 4 day seeds transcriptome and PEQU genome
hsp90_7_day_pequ.fas	alignment of the hsp70 genes from 7 day seeds transcriptome and PEQU genome
hsp90_sepal_pequ.fas	alignment of the hsp70 genes from sepal transcriptome and PEQU genome
hsp90_column_pequ.fas	alignment of the hsp70 genes from column transcriptome and PEQU genome
hsp90_lip_pequ.fas	alignment of the hsp70 genes from lip transcriptome and PEQU genome
hsp90_petal_pequ.fas	alignment of the hsp70 genes from petal transcriptome and PEQU genome
The HSP gene files were deposited in the Dryad Digital Repository (Data Citation 1). PEQU means *P. equestri*; flower bud, root, stem and leaf are labelled as fb, L5, L6 and PHA, respectively. The 12-, 7- and 4-day seeds were sown on 1/2 MS medium for 12, 7 and 4 days, respectively.	

**Table 8:** Quality control and data statistics of the raw reads.

**Type**	**L5_root**	**L6_stem**	**PHA_leaf**	**fb_flower bud**	**12_day seed**	**7_day seed**	**4_day seed**	**column**	**lip**	**petal**	**sepal**
Read number	49,848,468	66,141,114	15,999,780	70,571,268	53,861,172	53,200,618	52,791,758	53,212,746	51,175,078	54,004,470	51,191,360
Read length	90	90	75	90	90	90	90	90	90	90	90
Q20 (%)	95.8	94.1	88.9	94.5	99.9	99.9	99.9	99.8	99.9	99.8	99.7
GC percentage (%)	45	46	49	48	48	48	48	48	46	47	49

**Table 9:** Assembly statistics.

**Type**	**L5_root**	**L6_stem**	**PHA_leaf**	**fb_flower bud**	**12_day seed**	**7_day seed**	**4_day seed**	**column**	**lip**	**petal**	**sepal**
Total unigenes	107,406	106,002	26,051	49,443	35,466	30,995	29,428	47,303	53,045	36,674	32,669
Total transcripts	152,545	159,409	28,582	69,824	49,520	41,506	40,060	68,976	73,732	51,634	43,805
N50	787	1,298	742	1,575	1,321	1,222	1,370	1,165	1,063	1,311	1,245
Average length	576	764	584	911	849	824	911	762	703	874	844

**Table 10:** Mapping rates of the reads and transcript assembly completeness.

	**PHA_leaf**		**fb_flower bud**		**L5_root**		**L6_stem**		**lip**		**column**		**sepal**		**petal**		**4_day seed**		**7_day seed**		**12_day seed**	
	**count**	**percentage**	**count**	**percentage**	**count**	**percentage**	**count**	**percentage**	**count**	**percentage**	**count**	**percentage**	**count**	**percentage**	**count**	**percentage**	**count**	**percentage**	**count**	**percentage**	**count**	**percentage**
proper_pairs	10946586	86.99	50305282	88.42	30323300	85.32	41855338	85.83	42208656	92.89	44150392	93.78	45145406	94.75	46461164	93.72	45049842	94	42289318	86.36	30765662	94.1
CEGs	140	56.45	241	97.18	202	81.45	222	89.52	225	90.73	229	92.34	228	91.94	234	94.45	233	93.95	219	88.31	231	93.15
The mapping rate was tested by Bowtie mapping reads back to the unigenes. This table shows only the numbers and percentages of proper pairs. Count indicates the number of reads mapping back to the unigenes, and percentage indicates the read percentage. The transcript assembly completeness was assessed using CEGMA: count indicates the number of the 248 ultra-conserved CEGs present in the transcript assemblies, and percentage indicates the percentage of the 248 ultra-conserved CEGs present.																						

**Table 11:** Annotation statistics.

**Type**	**L5_root**	**L6_stem**	**PHA_leaf**	**fb_flower bud**	**12_day seed**	**7_day seed**	**4_day seed**	**column**	**lip**	**petal**	**sepal**
Unigene number	107,406	106,002	26,051	49,443	35,466	30,995	29,428	47,303	53,045	36,674	32,669
Nr	32,996	30,203	20,923	22,558	18,787	22,694	19,851	25,005	24,614	23,488	23,097
COG	8,823	8,243	6,633	8,283	8,802	9,194	8,886	9,874	9,549	9,746	9,518
KEGG	14,596	13,001	11,330	12,144	11,857	12,642	11,910	13,473	13,092	13,091	12,946

**Table 12:** Statistical results for the predicted CDSs.

**Number**	**fb_flower bud**	**L5_root**	**L6_stem**	**PHA_leaf**	**12_day seed**	**7_day seed**	**4_day seed**	**column**	**lip**	**petal**	**sepal**
Total	34,497	57,793	53,316	24,299	18,291	19,099	17,909	21,364	21,013	20,756	20,068

**Table 13:** YABBY gene families in the assembled transcriptomes.

**column**	**lip**	**petal**	**sepal**	**fb_flower bud**	**L6_stem**	**PHA_leaf**	**L5_root**	**4_day seed**	**7_day seed**	**12_day seed**
6	7	7	6	6	6	2	0	0	0	0

**Table 14:** NBS-encoding gene families in the assembled transcriptomes.

**column**	**lip**	**petal**	**sepal**	**fb_flower bud**	**L6_stem**	**PHA_leaf**	**L5_root**	**4_day seed**	**7_day seed**	**12_day seed**
17	17	14	18	24	22	13	21	12	7	17
